# Drugs for Autoimmune Inflammatory Diseases: From Small Molecule Compounds to Anti-TNF Biologics

**DOI:** 10.3389/fphar.2017.00460

**Published:** 2017-07-12

**Authors:** Ping Li, Ying Zheng, Xin Chen

**Affiliations:** State Key Laboratory of Quality Research in Chinese Medicine, Institute of Chinese Medical Sciences, University of Macau, Macau, China

**Keywords:** tumor necrosis factor alpha (TNF), autoimmune inflammatory diseases, non-steroidal anti-inflammatory drugs (NSAIDs), anti-TNF biologics, TNF receptor

## Abstract

Although initially described as an anti-tumor mediator, tumor necrosis factor-alpha (TNF) is generally considered as the master pro-inflammatory cytokine. It plays a crucial role in the pathogenesis of inflammatory diseases, such as rheumatoid arthritis (RA), inflammatory bowel disease, ankylosing spondylitis (AS), and psoriasis. Consequently, anti-TNF therapy has become mainstay treatment for autoimmune diseases. Historically, anti-inflammatory agents were developed before the identification of TNF. Salicylates, the active components of *Willow* spp., were identified in the mid-19th century for the alleviation of pain, fever, and inflammatory responses. Study of this naturally occurring compound led to the discovery of aspirin, which was followed by the development of non-steroidal anti-inflammatory drugs (NSAIDs) due to the chemical advances in the 19th–20th centuries. Initially, the most of NSAIDs were organic acid, but the non-acidic compounds were also identified as NSAIDs. Although effective in the treatment of inflammatory diseases, NSAIDs have some undesirable and adverse effect, such as ulcers, kidney injury, and bleeding in the gastrointestinal tract. In the past two decades, anti-TNF biologics were developed. Drugs belong to this class include soluble TNF receptor 2 fusion protein and anti-TNF antibodies. The introduction of anti-TNF therapeutics has revolutionized the management of autoimmune diseases, such as RA, psoriatic arthritis (PsA), plaque psoriasis (PP), AS, CD and ulcerative colitis (UC). Nevertheless, up to 40% of patients have no response to anti-TNF treatment. Furthermore, this treatment is associated with some adverse effects such as increased risk of infection, and even triggered the *de novo* development of autoimmune diseases. Such harmful effect of anti-TNF treatment is likely caused by the global inhibition of TNF biological functions. Therefore, specific inhibition of TNF receptor (TNFR1 or TNFR2) may represent a safer and more effective treatment, as proposed by some recent studies. In this review article, the historical development of anti-inflammatory drugs after World War II as briefly described above will be reviewed and analyzed. The future trend in the development of novel TNF receptor-targeting therapeutics will be discussed in the context of latest progress in the research of TNF biology.

## Introduction

Autoimmune inflammatory diseases affect approximately 7.6–9.4% of the world population, especially among the young and middle-aged women (Cooper et al., 2009; Bragazzi et al., 2016). Frequently accompanied by severe and chronic morbidity, autoimmune diseases are also leading causes of death all around world. The patients with autoimmune inflammatory diseases need intensive medical intervention, which imposes the huge burden on public health service and economy (Bragazzi et al., 2016). Excessive and prolonged activation of immune cells, such as T and B lymphocytes, and overexpression of the master pro-inflammatory cytokine tumor necrosis factor alpha (TNF), together with other mediators such as interlukin-6 (IL-6), interlukin-1 (IL-1), and interferon gamma (IFN-γ), play a central role in the pathogenesis of autoimmune inflammatory responses in rheumatoid arthritis (RA), inflammatory bowel disease (IBD), Crohn’s disease (CD), and ankylosing spondylitis (AS) (Moudgil and Choubey, 2011; Sticherling, 2016; Ellis and Braley-Mullen, 2017).

Non-steroidal anti-inflammatory drugs (NSAIDs), glucocorticoids, disease-modifying antirheumatic drugs (DMARDs) are traditionally used in the treatment of autoimmune inflammatory diseases. NSAIDs and glucocorticoids are effective in the alleviation of pain and inhibition of inflammation, while DMARDs have the capacity of reducing tissue and organ damage caused by inflammatory responses (Tabas and Glass, 2013). More recently, treatment for RA and other autoimmune diseases has been revolutionized with the discovery that TNF is critically important in the development of the diseases (Monaco et al., 2015). Anti-TNF biologics (such as infliximab, adalimumab, etanercept, golimumab, and certolizumab pepol) have markedly improved the outcome of the management of autoimmune inflammatory diseases (Meier et al., 2013). However, a considerable proportion of patients do not respond to anti-TNF treatment (Roda et al., 2016). Moreover, anti-TNF biologics are expensive, and are associated with some adverse effects. Recent studies indicate that specifically targeting of one of TNF receptors may represent a more effective and safer treatment for autoimmune disorders. In this review, the history of development of anti-inflammatory drugs, from small molecules to anti-TNF antibodies, will be discussed.

## Non-Steroidal Anti-Inflammatory Drugs (NSAIDs)

### The Historical Development of NSAIDs

Plant parts such as leaves of myrtle (Myrtus), bark of willow tree (Salix), bark of poplar (Populus), and meadow sweet (Spirea) were used in fork medicine for centuries in the treatment of fever, pain, and inflammatory responses (Hedner and Everts, 1998). These medicinal plants represented a primitive form of anti-inflammatory drugs. In the mid-19th century, salicylates were identified as active components of *Willow* spp. responsible for the anti-inflammatory activity, which laid the foundation for the mass synthesis of acetylsalicylic acid in 1899 (Vainio and Morgan, 1997; Vane, 2000). The progress in chemistry in the 19th–20th centuries promoted the fast development of NSAIDs. Initially, most of NSAIDs were the organic acid, but the non-acidic compounds were also discovered later. With outstanding safety profiles at dose ranges, ibuprofen was the first NSAIDs approved in the United Kingdom (Busson, 1986). After that, pharmaceutical companies began to develop NSAIDs with a series of chemical and biological properties (Rainsford, 2007). Overall, Post-World War II, the development of NSAIDs had experienced two periods: one was the pre-prostaglandin period (∼1970s) and another one was from 1970s to the end of the last century in which drugs were screened and evaluated partially based on their effect on the production of prostaglandin (Rainsford, 2007).

### The Discovery of NSAIDs

Salicylic acid was synthesized by the Gerland in 1853 for the first time, and acetylsalicylic acid was synthesized by Charles Gerhardt in 1853 (Gerhardt, 1853; Gerland, 1853). Until 1876, salicylic acid was firstly used in clinic for the treatment of rheumatic disorders by two German physicians, Drs Streicher and Reiss (Hedner and Everts, 1998). Acetylsalicylic acid was re-discovered by Hoffman in 1897 (Hoffmann and Förster, 1987), and it became available worldwide in the treatment of rheumatic disorders and pain since then (Hedner and Everts, 1998). Acetyl-salicylate was first used as Aspirin in 1899 (Vainio and Morgan, 1997). The development of aspirin, a prototype of NSAIDs, was a landmark (Vainio and Morgan, 1997), which was followed by the development of phenylbutazone (1946) and indomethacin (1963) (Shen, 1982). The term of ‘non-steroidal anti-inflammatory drug’ was used for the first time when phenylbutazone was introduced 3 years later as an anti-inflammatory agent. Thus, aspirin, phenylbutazone, and indomethacin were founding members of NSAID family.

### The Categories of NSAIDs

Non-steroidal anti-inflammatory drugs have the analgesic, antipyretic, and anti-inflammatory effect, frequently used for the treatment of conditions like arthritis and headaches (Rainsford, 2007). NSAIDs relieve pain through blocking cyclooxygenase (COX) enzymes (Simmons et al., 2004). COX promotes the production of prostaglandins, a mediator which causes inflammation and pain (Simmons et al., 2004). Although NSAIDs have different chemical structures, all of them have the similar therapeutic effect, e.g., inhibition of autoimmune inflammatory responses (Rainsford, 2007). In general, NSAIDs can be divided into two broad categories: traditional non-selective NSAIDs and selective cyclooxygenase-2 (COX-2) inhibitors (Antman et al., 2007).

#### Traditional Non-selective NSAIDs

Based on the chemical structure, the traditional non-selective NSAIDs can be classified into different sub-types (Antman et al., 2007): (1) salicylic acid derivatives: acetylsalicylic acid (aspirin), diflunisal and sulfasalazine; (2) para-aminophenol derivatives: acetaminophen; (3) fenamates: mefenamic acid, meclofenamate, flufenamic acid; (4) propionic acid derivatives: ibuprofen, naproxen, fenoprofen, ketoprofen, flurbiprofen, oxaprozin; and (5) enolic acid (oxicam) derivatives: piroxicam, tenoxicam. Most of these drugs were discovered in the pre-prostaglandins period and were developed in the 1960s. Their antipyretic, analgesic, and anti-inflammatory properties were discovered by animal studies, based on some biochemical experimental systems (Rainsford, 2007).

#### Selective COX-2 Inhibitors

The anti-inflammatory effect of NSAIDs is mainly based on the inhibition of activity of cyclooxygenase (COX) enzymes (Simmons et al., 2004). COX enzymes have two forms, e.g., COX-1 and COX-2. The COX-1 is constitutively expressed by the most of tissues and is responsible for the formation of prostaglandins and thromboxane A2, while the expression of COX-2 needs to be induced by inflammatory mediators (Antman et al., 2007). COX-1 plays an important role in some important physiological processes (Crofford, 1997). Therefore, complete inhibition of both COX-1 and COX-2 inevitably results in severe side effects (Cheng and Visco, 2012).

Inhibition of COX-1 by traditional non-selective NSAIDs causes various gastrointestinal toxicities. In order to reduce this harmful side effect, the selective COX-2 inhibitors were developed (Grosser et al., 2006). The selective COX-2 inhibitors can be further divided into two categories: selective COX-2 inhibitors and highly selective COX-2 inhibitors. The selective COX-2 inhibitors include meloxicam, salicylate, and nimesulide. These drugs more selectively inhibit COX-2, as compared to their action on COX-1. The highly selective COX-2 inhibitors characterized by replacing *cis*-stilbene with one of the pendant phenyl rings by different substitutes, termed as diarylheterocycles (Khanapure et al., 2003), including celecoxib, rofecoxib, valdecoxib, lumiracoxib, parecoxib, and etoricoxib (Antman et al., 2007). These coxibs have the same structure of diarylheterocycles which is decisively important for their highly potent inhibitory effect on COX-2 (Rainsford, 2007).

### The Side Effects of NSAIDs

The traditional non-selective NSAIDs are able to inhibit both COX-1 and COX-2. These drugs also inhibit platelet aggregation and cause significant gastrointestinal disorders such as bleeding, ulcers, and perforation (Fujita et al., 2013). They also induce renal toxicity (Murray and Brater, 1993; Fujita et al., 2013). In contrast, the gastrointestinal adverse effect of selective COX-2 inhibitors is markedly reduced (Dajani and Islam, 2008). However, just like traditional non-selective NSAIDs, selective COX-2 inhibitor still have adverse effects on the cardiovascular system, including congestive heart failure, acute myocardial fraction, and even sudden death (Ray et al., 2002; Hermann and Ruschitzka, 2006).

## Glucocorticoids

The glucocorticoids were discovered by Hench and Kendal in 1940s (Hench et al., 1949), through an observation that cortisone had a significant beneficial effect for patients with severe RA (Glyn, 1998; Ferreira et al., 2016). The synthetic development and commercial efforts on glucocorticoids (or corticosteroids) were made in 1950–1980s, and consequently, a number of drugs were yielded (Neeck, 2002). Since then, the synthetic glucocorticoids are extensively used in the treatment of RA, asthma and other inflammatory diseases (Rhen and Cidlowski, 2005). These drugs include prednisone/prednisolone, methylprednisolone, and the fluorinated glucocorticoids such as dexamethasone and betamethasone, which are more frequently used (Buttgereit et al., 2011). Prednisone is an inactive pro-drug and its active metabolite is prednisolone (Frey, 1987). Usually, the prednisolone is administered once a day, while the duration of action of other glucocorticoids is longer when administered into patients with rheumatic diseases (Laev and Salakhutdinov, 2015). The binding of glucocorticoids to its specific receptor results in the inhibition of cellular signaling pathway such as AP-1 and NF-κB, and consequently regulate the expression of cytokines and chemokines (Barnes, 1998). We for the first time found that immunosuppressive CD4^+^Foxp3^+^ regulatory T cells (Tregs) were relatively more resistant to glucocorticoid-induced cell death (Chen et al., 2004). Furthermore, glucocorticoid selectively inhibited the proliferation of effector T cells (Teff) while promoting Treg proliferation induced by IL-2 (Chen et al., 2006). This property of glucocorticoid was harnessed to induce immune tolerance which may represent a novel approach in the treatment of autoimmune diseases (Kang et al., 2008).

Although the glucocorticoids are highly effective in the treatment of chronic diseases, they also induce severe side effects, including gastrointestinal ulcers and bleeding, infection, immunosuppression, and bone damage (Ethgen et al., 2013). Currently, low-doses of glucocorticoids are usually used in the treatment of autoimmune inflammatory diseases, while the adverse effects remain a major concern for prolonged usage (Curtis et al., 2006; Rainsford, 2007).

## Conventional Disease-Modifying Anti-Rheumatic Drugs (cDMARDs)

To relieve pain is a major goal in the management of RA and other arthritis. NSAIDs are able to alleviate pain and improve joint function through inhibition of inflammatory responses (Rainsford, 2007). However, NSAIDs alone do not reverse the pathological course of the autoimmune diseases (Tabas and Glass, 2013). Glucocorticoids can partially reduce pain symptom and suppress inflammation in the short-term (Laev and Salakhutdinov, 2015). Therefore, drugs that are capable of sustainably reducing inflammation with manageable side effects are highly desirable for the treatment of RA and other autoimmune diseases (Laev and Salakhutdinov, 2015). For this purpose, several drugs with different chemical structures, known as “disease-modifying anti-rheumatic drugs” (DMARDs), were developed.

### Methotrexate

Methotrexate is an analog and antagonist of folic acid, which was synthesized firstly in 1947 (Swierkot and Szechinski, 2006). Methotrexate was firstly reported to be effective in the treatment of RA in 1962 (Kersley, 1968), and after that it was also widely used as an anti-inflammatory drug for the management of other autoimmune diseases, including psoriasis, CD, and UC (Nathan et al., 2008; Yelamos and Puig, 2015). Methotrexate has been used as the first-line DMARDs since it was developed, due to its long-term effectiveness, satisfying responsive rate, acceptable toxicity profile, and low cost (Visser and van der Heijde, 2009; Favalli et al., 2014). Fundamental mechanisms underlying the therapeutic effect of methotrexate have been elucidated. For example, methotrexate was found to competitively inhibit the activity of folate-dependent enzymes, and synthesis of purine and pyrimidine which are required for DNA and RNA synthesis, and consequently suppress lymphocyte proliferation and production of pro-inflammatory cytokines (Meier et al., 2013). Nevertheless, the methotrexate monotherapy is no longer considered as the gold standard for RA treatment, for the reason that DMARDs combination therapy is more effective (Kremer et al., 2004).

### Leflunomide

Leflunomide was approved for the treatment of RA in the 1998 (Gabriel et al., 2001). Leflunomide is an isoxazole derivative that is capable of blocking the rate-limiting enzyme dihydroorotate dehydrogenase, thereby selectively inhibits the synthesis of *de novo* pyrimidine ribonucleotides such as rUMP (Fox et al., 1999). Leflunomide is a pro-drug that is metabolized into teriflunomide *in vivo* (Laev and Salakhutdinov, 2015). Teriflunomide is a potent inhibitor of NF-κB activation, and consequently suppresses the production of pro-inflammatory cytokines. Leflunomide also reduces the production of metalloproteinases in synovial tissue and prevents joint destruction (Laev and Salakhutdinov, 2015). The results of meta-analyses strongly suggest that leflunomide is a DMARDs and effective in the treatment of RA (Osiri et al., 2003). As shown by several studies, the therapeutic effect of leflunomide is comparable to that of methotrexate (Silverman et al., 2005). Leflunomide can improve all clinical outcomes and delay radiographic progression at 6 and 12 months of RA patients (Sharp et al., 2000). Therefore, leflunomide should be considered as first-line therapy if contraindications to methotrexate are present. Furthermore, leflunomide is very useful in the combination therapy (Kalden et al., 2005).

### Other DMARDs

Except for methotrexate and leflunomide, other DMARDs, include gold compounds, sulfasalazine, azathioprine, cyclophosphamide, antimalarials, D-penicillamine, cyclosporine, are all effective in the treatment of autoimmune inflammatory diseases such as RA (Gabriel et al., 2001). The DMARDs commonly used for the treatment of RA include hydroxychloroquine, sulfasalazine, methotrexate and leflunomide, while D-penicillamine, azathioprine, gold compounds, and cyclosporine are used less frequently (Kim et al., 2013).

Parenteral organic gold compounds have been used for RA treatment since 1920s (Simon, 2004), and their effect is comparable with other DMARDs (Meier et al., 2013). Nonetheless, the toxicity of gold compounds, especially the adverse effect on skin and mucosa, is a major concern which limits their clinical application (Bendix and Bjelle, 1996).

Sulfasalazine was initially investigated for the treatment of IBD, including UC and CD (Box and Pullar, 1997). This drug was firstly reanimated in Europe where it has been used in early RA patients. The exact mechanism of sulfasalazine is still unclear, although it inhibits the production of pro-inflammatory cytokines, including TNF and IL-6 (Wahl et al., 1998). The main side effect of sulfasalazine is gastrointestinal upset or rash (Skosey, 1988).

Antimalarial drugs hydroxychloroquine and chloroquine have the capacity to inhibit the clinical progress of several autoimmune diseases, including RA and systemic lupus erythematosus (SLE) (Taherian et al., 2013). Although the exact molecular mechanism of its immunosuppressive effect remains to be fully understood, both drugs can inhibit the activation of T cells, B cells (Taherian et al., 2013) and the production of pro-inflammatory cytokines such as TNF, IL-6, and IL-1β (Jang et al., 2006). Both drugs almost have the same side effects, such as rare irreversible retinopathy and a more rarely form of myopathy (Costedoat-Chalumeau et al., 2015).

### Combination Therapy with cDMARDs

Among cDMARDs drug class, the methotrexate is still an ‘anchor drug’ for RA therapy, and the first choice for the most of DMARD-naïve patients (Favalli et al., 2014). However, a considerable proportion of patients do not respond to methotrexate. Either anti-TNF biologics (which will be discussed later) or a combination therapy of cDMARDs should be considered for those patients (van Vollenhoven et al., 2012). Methotrexate has been used in combination with other drugs, including sulfasalazine, sulfasalazine and hydroxychloroquine, cyclosporine and biologic agents such as etanercept and infliximab (Kremer et al., 2004; Swierkot and Szechinski, 2006). For example, the concomitant treatment of methotrexate and leflunomide results in a synergistic effect in the treatment of patients with RA (Boers et al., 1997; Kremer et al., 2004). The triple combination of methotrexate, sulfasalazine and hydroxychloroquine represent an effective therapy regimen since 1996 (Meier et al., 2013), which is more effective and safer than the monotherapy with methotrexate, or methotrexate combination of sulfasalazine, or hydroxychloroquine (ODell et al., 1996, 2002). It has been reported that the combination of methotrexate and cyclosporine, or leflunomide and cyclosporine, are more effective than each drug alone (Karanikolas et al., 2006). However, the toxicity of cyclosporine, including reversible and irreversible renal disease, hypertension and heurism, remains an important issue in the combination therapy (Gabriel et al., 2001). Therefore, the ideal cDMARDs combination therapy should be one that is synergistic in their efficacy while lacking additive effects of toxicity.

## Anti-Tnf Biologics

In 1975, Lloyd and colleagues discovered an endotoxin-inducible serum factor named TNF had the capacity to cause necrosis of tumors (Carswell et al., 1975). It is one of subsequent studies of “Coley’s toxin,” a therapeutic regimen using killed bacteria to induce tumor regression in cancer patients, invented by Dr. William Coley in 1891 (Coley, 1891). Although initially identified as anti-tumor molecule, TNF is now considered as a pleiotropic cytokine which plays a major role in immune or inflammatory responses (Palladino et al., 2003; Efimov et al., 2009).

Feldman’s group showed that a number of pro-inflammatory cytokines such as IL-1, TNF, IL-6, GM-CSF, and IFN-γ were expressed at a high levels in synovium samples from active RA patients (Feldmann et al., 1996). Intriguingly, anti-TNF antibody inhibited the expression of other pro-inflammatory cytokines (IL-1, IL-6, and GM-CSF) in the synovial culture (Feldmann et al., 1996). Furthermore, anti-TNF antibodies such as infliximab and adalimumab suppressed antigen-induced IFN-γ production in blood (Wallis, 2007). These results provide the evidence that TNF plays a predominant role in the pathogenesis of autoimmune inflammatory diseases. IFN-γ is also an important mediator in the inflammatory responses, such as Schwartzman reactions (Billiau, 1988) and collagen-induced arthritis (Mauritz et al., 1988). It was also reported that IFN-γ promoted the production of TNF induced by LPS stimulation (Billiau, 1988). However, unlike the effect of anti-TNF biologics, treatment with antibody against IFN-γ did not markedly ameliorate the arthritic symptoms in RA patients (Sigidin et al., 2001; Schurgers et al., 2011).

TNF is now generally considered as a master pro-inflammatory cytokine that plays a critical role in the pathogenesis of autoimmune inflammatory diseases (Moudgil and Choubey, 2011). Consequently, anti-TNF biologics, which are designed to block the biological function of TNF, have been developed for the therapy of autoimmune inflammatory diseases (Meier et al., 2013). In the past two decades, five TNF-targeting drugs have been approved for clinical use, including infliximab, etanercept, adalimumab, golimumab, and certolizumab pegol, for the treatment of the autoimmune inflammatory diseases including RA, CD and AS (Monaco et al., 2015).

### Infliximab

Infliximab is a recombinant chimeric antibody that is generated by mouse myeloma cells. The structure of infliximab contains a constant sequence of human IgG1 and variable regions of murine, which is specific for all forms of TNF in human and chimpanzees, and effectively blocks the binding of TNF to its soluble and membrane receptors (Monaco et al., 2015). The maximum serum concentration of infliximab can be reached within 1 h by intravenous administration (Atzeni et al., 2005), and the half-life is approximately 8–10 days (Chatzantoni and Mouzaki, 2006). After that, infliximab can be maintained by dosing every 8 weeks. Infliximab is capable of mediating both complement-dependent and antibody-dependent cytotoxicity on a cell line overexpressing TNF (Tracey et al., 2008). Infliximab is initially approved for the treatment of Crohn’s disease by the US Food and Drug Administration (FDA) and later also for RA. It was further approved for the treatment of AS, PsA, UC, CD, and chronic plaque psoriasis (Atzeni et al., 2005).

### Etanercept

Etanercept comprises soluble TNF receptor 2 and Fc portion of IgG1, which is a human recombinant protein (Kerensky et al., 2012). After subcutaneous administration, etanercept has a half-life of 3–3.5 days (Chatzantoni and Mouzaki, 2006). Etanercept binds to and inactivates soluble form and membrane form of TNF and lymphotoxin, by blocking their interaction with the receptors (Tracey et al., 2008). Similar as infliximab, etanercept alleviate the signs and symptoms of arthritis and inhibits progress of RA in patients (Moreland et al., 1999). Furthermore, it is more effective when combined with methotrexate (Emery et al., 2008). Etanercept is also approved by FDA for the treatment of juvenile rheumatoid arthritis and PsA, RA, PP, and AS (Kerensky et al., 2012).

### Adalimumab

Adalimumab is a fully humanized IgG1 monoclonal antibody, which can specific block human TNF binding to its receptors (Atzeni et al., 2005). Adalimumab needs a less frequent subcutaneous injection because of its relative longer half-life which is about 2 weeks (Simon, 2004). Furthermore, adalimumab shows the lower immunogenicity than infliximab (Rau, 2002).

Adalimumab is effective in alleviating the signs and symptoms of moderate to severe RA patients (van de Putte et al., 2004). Adalimumab is also effective in the treatment of patients with CD, with good tolerance and minimal immunogenicity, thus can be used in patients with allergic reactions to infliximab (Sandborn et al., 2004). Adalimumab has been approved by FDA for the treatment of autoimmune diseases including RA, PsA, AS, and CD (Lapadula et al., 2014).

### Golimumab

Golimumab is also a fully humanized IgG1 monoclonal antibody, which has a high affinity and specificity for human TNF (Shealy et al., 2007). As shown by the preclinical data, golimumab has higher affinity and is more effective in the neutralization of soluble and trans-membrane forms of TNF, as compared with infliximab and adalimumab, thus it can potently neutralize TNF biological activity (Shealy et al., 2010). The half-life of golimumab is about 7–20 days (Zhou et al., 2007). Golimumab was approved by the FDA in 2009 for the treatment of moderate-to-severe RA when administrated in combination with methotrexate (Mazumdar and Greenwald, 2009). It was also approved for UC treatment by FDA and European Medicines Agency (EMA) in 2013 (Lowenberg and D’Haens, 2013).

### Certolizumab Pegol

Certolizumab pegol (CDP870) is a humanized monoclonal antibody with the structure of polyethylene glycolated Fab fragment, which is a novel TNF inhibitor with a distinct mechanism of action compared with other TNF inhibitors (Pasut, 2014). The unique structure might be an explanation for certolizumab pegol with higher efficiency in comparison with other TNF inhibitors (Desai et al., 2012). PEGylation of certolizumab pegol improves its half-life to 2 weeks which may contribute to its high concentration in the inflamed tissues (Keystone et al., 2008).

Certolizumab pegol monotherapy could effectively control the symptoms of patients with RA (Choy et al., 2002). It is also effective the treatment of patients with moderate to severe CD (Schreiber et al., 2005). Certolizumab pegol has been approved for the treatment of CD and PsA by FDA (Deeks, 2016).

### Biosimilars

Biosimilar has been defined as a biological medicinal product that has the similar quality, safety, and efficacy with the already approved biological medicine (Fiorino and Danese, 2014). Anti-TNF biosimilars that are extensively developed in recent years will help reduce the cost of anti-TNF treatment. To date, a number of anti-TNF biosimilar products (summarized in **Table 1**) have been marketed (Dorner and Kay, 2015). CT-13 is the first biosimilar monoclonal antibody against TNF. Comparing with the original TNF inhibitor infliximab, CT-13 is equally effective and safe (Yoo et al., 2013). It is approved in European for the treatment of RA, AS, PsA, PS, CD, and UC (Dorner et al., 2016).

**Table 1:** Anti-TNF biosimilars in the development for the treatment of autoimmune inflammatory diseases.

Anti-TNF biologics	Biosimilar	Indicates	Current development stage in May 2017	Sponsor
Infliximab	ABP 710	RA	Phase III in RA	Amgen
	BCD-055	RA, Psoriasis, AS	Phase III in RA, Psoriasis Phase I completed in AS	Biocad
	BOWO15	RA	Phase III in RA	Epirus Biopharmaceuticals (Switzerland) GmbH
	CT-P13	RA,CD,AS	Phase III completed in CD, RA Phase III completed in AS	Celltrion
	NI-071	RA	Phase III completed in RA	Nichi-Iko Pharmaceutical Co., Ltd.
	PF-06438179	RA	Phase III in RA	Pfizer
	SB2	RA	Phase III completed in RA	Ohio State University Comprehensive Cancer Center
Etanercept	CHS-0214	RA,PP	Phase III completed in RA and PP	Coherus Biosciences, Inc.
	ENIA11	RA	Phase III in RA	TSH Biopharm Corporation Limited
	GP2015	RA,PP	Phase III completed in RA, Chronic Stable Plaque Psoriasis	Sandoz
	HD203	RA	Phase III completed in RA	Hanwha Chemical
	LBEC0101	RA	Phase III in RA	LG Life Sciences
	SB4	RA	Phase III completed in RA	Samsung Bioepis Co., Ltd.
Adalimumab	ABP 501	RA, Psoriasis	Phase III completed in RA and Psoriasis	Amgen
	BCD-057	Psoriasis	Phase III in Psoriasis	Biocad
	BI 695501	RA	Phase III completed in RA	Boehringer Ingelheim
	CHS-1420	PP	Phase III completed in PP	Coherus Biosciences, Inc.
	GP2017	RA, Psoriasis	Phase III completed in Plaque Type Psoriasis Phase III in RA	Sandoz
	LBAL	RA	Phase III in RA	LG Life Sciences
	M923	RA	Phase III completed in RA	Momenta Pharmaceuticals, Inc.
	PF-06410293	RA	Phase III in RA	Pfizer
	MSB11022	RA, PsA, PP	Phase III in RA, PsA, PP	EMD Serono Research & Development Institute, Inc.
	SB5	RA	Phase III completed in RA	Samsung Bioepis Co., Ltd.
Golimumab	BOW100	AS,PsA,RA,UC	Preclinical	Bioceros
	ONS-3035	RA, UC	Preclinical	Oncobiologics
Certolizumab pegol	PF688	RA, CD	Preclinical	Pfenex
	Xcimzane	RA, PsA, UC, AS	Preclinical	Xbrane

### The Side Effects of Anti-TNF Biologics

Although anti-TNF biologics are generally effective in the treatment of patients with different autoimmune inflammatory diseases (Willrich et al., 2015), not all patients respond equally well to the treatment. Up to 40% of patients have no response to anti-TNF treatment (Roda et al., 2016). Furthermore, several types of adverse effects have been associated with anti-TNF biologics.

It was reported recently that PBMCs isolated from patients responding to the treatment of adalimumab and etanercept can produce higher levels of TNF and soluble TNFR2 (sTNFR2) than those from patients responding to infliximab, and PBMCs isolated from patients who do not respond to infliximab produce higher levels of TNF and sTNFR2 than those from patients responding to infliximab (Gibellini et al., 2016). Therefore, anti-TNF biologics may have major and different effects on TNF expression and soluble TNFR2 levels in patients. It was reported that the TNF gene polymorphism (TNF-alpha 308G > A) is association with patients who do not respond to anti-TNF therapy (Wijnen et al., 2014). Furthermore, the polymorphism of tumor necrosis factor receptor superfamily member 1B, a gene encoding TNFR2 protein, is able to predict responses of patients with autoimmune diseases to anti-TNF therapy (Chen W. et al., 2015). Thus, aberrant expression of TNF and TNFR2, and their responses to TNF blockade, is responsible at least partially for the outcome of anti-TNF therapy.

TNF plays a crucial role in host defense to invading pathogens (Pfeffer, 2003). Anti-TNF biologics can inhibit IFN-γ expression in the blood (Wallis, 2007). In addition, TNF is a good inducer of nitric oxide (NO) in macrophage, and TNF also synergizes with IFN-γ in inducing inducible nitric oxide synthase (iNOS) (Vila-Del Sol et al., 2007), while iNOS is able to synthesize antimicrobial nitric oxide (Fang, 1997). This can explain why infections, such as tuberculosis and pneumonia, are common adverse event of patients treated with anti-TNF biologics (Antoni and Braun, 2002).

There is also some evidence that anti-TNF treatment is associated with increased risk of malignancy (Bongartz et al., 2006). However, this is not supported by the more recent studies (Minozzi et al., 2016; Shelton et al., 2016). In fact, the underlying inflammatory condition is likely to promote the development of cancer, while anti-inflammatory treatment has the beneficial effect in inhibition of malignancy (Lasry et al., 2016). For example, patient with RA and CD has a higher risk of lymphoma compared with the general population (Antoni and Braun, 2002). Therefore, patients with malignancy after treatment with anti-TNF biologics should be more carefully studied.

Anti-TNF treatment has been tried in patients with congestive cardiac failure, since the high levels of circulating TNF was found in such patients. However, infliximab, adalimumab, and etanercept have deleterious, rather than beneficial, effects on congestive cardiac failure patients (Balakumar and Singh, 2006). Anti-TNF biologics have been reported to have side effects on the neurological system. For example, anti-TNF treatment exacerbated diseases in almost all multiple sclerosis (MS) (Robinson et al., 2001). Paradoxically, some new autoimmune diseases can be *de novo* induced by anti-TNF therapy. For example, psoriasis can be induced in IBD patients treated with anti-TNF biologics (Guerra et al., 2012). Therefore, more effective and safer TNF-targeting treatment needs to be developed. Thorough understanding of the fundamental biology of TNF and it receptors is a prerequisite to reach this goal.

## Anti-TNFR Therapeutics: Next Generation of TNF-Targeting Drug?

Tumor necrosis factor-alpha is a pleiotropic cytokine that is involved in the initiation and orchestration of inflammation and immunity (Aggarwal, 2003). Two receptors of TNF, namely TNFR1 (P55) and TNFR2 (P75), mediate different signaling pathways and induce diverse biological effects of TNF (Vandenabeele et al., 1995). TNFR1 is expressed by almost all cell types except erythrocyte, whereas TNFR2 is more strictly expressed by immune cells. The capacity of TNF to induce cell death is mediated by TNFR1. Conversely, TNFR2 signaling triggers cell survival pathways and promotes cell proliferation (Faustman and Davis, 2010). Initially, TNF is expressed on the cell surface as a transmembrane protein. It is then cleaved by a metalloprotease, called TNF-alpha converting enzyme (TACE) (Black et al., 1997). This process liberates a trimeric soluble protein, namely soluble TNF (sTNF). Both membrane-bound TNF and soluble TNF can bind to TNFR1 and TNFR2, but membrane-bound TNF preferentially binds with TNFR2 (Grell et al., 1995).

There is now compelling evidence that TNFR2 is constitutively expressed by immunosuppressive CD4^+^Foxp3^+^ regulatory T cells (Tregs) and TNF-TNFR2 interaction preferentially activates and expands naturally occurring Tregs (Chen et al., 2007, 2008, 2010, 2013; Nguyen and Ehrenstein, 2016). The immunosuppressive property of TNFR2 has been shown by many studies. For example, TNFR2 agonist is able to promote the expansion of Tregs more potently than TNF itself, and consequently inhibits autoimmune responses (Okubo et al., 2013). Selective blockade of TNFR1 also results in the proliferative expansion and activation Tregs (McCann et al., 2014), and inhibits clinical symptoms in CIA model (Shibata et al., 2009). It was shown recently that therapeutic anti-TNF antibody Infliximab binds to and promotes the expression of membrane bound TNF on monocytes from RA patients, and consequently enhances Treg activity through TNF-TNFR2 interaction (Chen and Oppenheim, 2016; Nguyen and Ehrenstein, 2016). These evidences favor the idea that biologic drugs which can selectively inhibit TNFR1, or selectively activate TNFR2, may be more effective and safer than those globally inhibit TNF. The expression of TNFR2 on cells and tissues is more limited than TNFR1. Thus, therapeutically targeting TNFR2 may have fewer side effects than targeting TNFR1. Expression of TNFR2 on CD4^+^Foxp3^-^ effector T cells (Teffs) can be induced and upregulated by TCR stimulation, and TNFR2 expression on Teff cells has a functional consequence (Chen and Oppenheim, 2011; Govindaraj et al., 2013; Chen X. et al., 2015; Chen and Oppenheim, 2016; Chen et al., 2016; Zaragoza et al., 2016). Thus, the effect of TNFR2 on Teff function should be considered in the development of TNFR2-targeting therapeutics.

## Other Biological Drugs for Autoimmune Inflammatory Diseases

In addition to anti-TNF agents, the biologics targeting other proinflammatory cytokines or immune competent molecules have also been extensively studied and actively developed. For example, abatacept, a fully humanized fusion protein of extracellular domain of CTLA-4 and Fc fraction of IgG1, has been approved for the RA patients with inadequate response to anti-TNF therapy (Genovese et al., 2005). The major immunological mechanism of abatacept is selective inhibition of co-stimulation pathway (CD80 and CD86) and activation of T cells. Tocilizumab, a humanized anti-IL-6 receptor monoclonal antibody was approved for RA patients intolerant to DMARDs and/or anti-TNF biologics (Okuda, 2008). This therapeutic mAb blocks the transmembrane signaling of IL-6 through binding with soluble and membrane forms of IL-6 receptor. Biological drugs targeting IL-1 (anakinra), Th1 immune responses (IL-12/IL-23, ustekinumab), Th17 immune responses (IL-17, secukinumab) and CD20 (rituximab) have also been approved for the treatment of autoimmune diseases, as summarized in the **Table 2**, as a complementary and alternative biological treatment of anti-TNF therapy.

**Table 2:** Alternative biologic in autoimmune inflammatory diseases.

Drug	Target	Type of molecules	Indications	Reference
Abatacept (Orencia)	CD80 (B7-1) and CD86 (B7-2) and blocks activation of T-cell Ab4Ig	The extracellular domain of CTLA4 and Fc domain of IgG1	RA, JIA, SLE	Genovese et al., 2005
Tocilizumab (Actemra)	IL-6	A humanized anti-human IL-6 receptor monoclonal antibody	RA after treatment failure with TNF inhibitors	Okuda, 2008
Anakinra (Kineret)	IL-1	A recombinant human IL-1 receptor antagonist	RA	Fleischmann et al., 2003
Ustekinumab	IL-12 and IL-23	Human IgG1k mAb	Psoriasis	Koutruba et al., 2010
Rituximab	CD20	A chimeric murine/human monoclonal IgG1k antibody	RA, SLE	Fleischmann, 2009
Secukinumab	IL-17	A human IgG1κ monoclonal antibody	PP	Campa et al., 2016

*CTLA, cytotoxic T lymphocyte-associated; JIA, juvenile idiopathic arthritis; SLE, systemic lupus erythematosus.*

## Conclusion

Although TNF plays a central role in acute and chronic inflammation, the anti-inflammatory agents were developed before the identification of TNF. After the World War II, agents from small molecules to anti-TNF antibodies have been developed, including NSAIDs, glucocorticoids, DMSADs, and anti-TNF biologics. The traditional non-selective NSAIDs are associated with severe gastrointestinal disorders, attributable to simultaneous inhibition of COX-1 and COX-2, which prompted the development of selective NSAIDs. Glucocorticoids have potent anti-inflammatory activity, accompanied by some severe adverse effects. Methotrexate remains the first-line drug, while the efficacy can be enhanced and adverse effects can be reduced by DMARDs combination therapy. Currently, five anti-TNF biologics have been approved for patients with autoimmune disorders. With great success in treating autoimmune diseases, anti-TNF biologics also represent the most profitable drug class in the history, exceeding $US 25 billion total sale in 2012 (Monaco et al., 2015). Anti-TNF biosimilars bring the hope to reduce the medical costs and consequently improve the accessibility to this revolutionized treatment. Nevertheless, the effectiveness and safety of TNF-targeting treatment should be further improved, hopefully through the selective blockade of TNFR1 or activation of TNFR2. For those patients failed to respond appropriately to anti-TNF treatment, biologics targeting other pro-inflammatory cytokines and pathways provide an alternative therapy.

As compared to small molecule compounds, biologics have some clear advantages, such as higher safety profile or minimal toxicity, well-understood mechanisms, and more importantly, the target specificity (Tracey et al., 2008; Meier et al., 2013; Monaco et al., 2015). Due to these advantages, the biologics, including therapeutic monoclonal antibodies and antibody-drug conjugate (ADC), have become main stream therapeutics (Meier et al., 2013; Monaco et al., 2015). This class of drug represents the fastest growing segment of global pharmaceutical marked (based on the data of global sale and FDA approval) (Ioannidis et al., 2013; Monaco et al., 2015). Therefore, it is predictable that the development of new biologics for the treatment of autoimmune diseases will be the focus in the future, which will be facilitated by the more thorough understanding of molecular basis of autoimmune diseases.

## Author Contributions

PL drafted the work; YZ and XC revised the manuscript. PL, YZ, and XC contributed substantially to the conception or design of the work, approved the final version to be published, and agreed to be accountable for all aspects of the work in ensuring that questions related to the accuracy or integrity of any part of the work are appropriately investigated and resolved.

## Conflict of Interest Statement

The authors declare that the research was conducted in the absence of any commercial or financial relationships that could be construed as a potential conflict of interest.
